# Maternal tobacco exposure during pregnancy and allergic rhinitis in offspring: A systematic review and meta-analysis

**DOI:** 10.1097/MD.0000000000026986

**Published:** 2021-08-27

**Authors:** Yaqian Zhou, JunRong Chen, Yunpeng Dong, Jinhua Shen, Mei Tian, Yide Yang, Liujiang Song, Jian Li

**Affiliations:** a Key Laboratory of Model Animals and Stem Cell Biology in Hunan Province, School of Medicine, Hunan Normal University, Hunan, China; b Department of Otolatyngoloty-Head and Neck Surgery, Yichang Central People's Hospital, the First College of Clinical Medical Science, Three Gorges University, Hubei, China; c Key Laboratory of Molecular Epidemiology of Hunan Province, School of Medicine, Hunan Normal University, Hunan, China; d Gene Therapy Center, University of North Carolina at Chapel Hill, North Carolina, USA; e Key Laboratory of Study and Discovery of Small Targeted Molecules of Hunan Province, School of Medicine, Hunan Normal University, Hunan, China.

**Keywords:** allergic rhinitis, offspring, prenatal tobacco exposure

## Abstract

**Background:**

Maternal tobacco exposure during pregnancy is known to cause a potential hazard to the offspring's health. So far, published studies have shown no consistent results with whether tobacco exposure in utero is causally linked to the development of allergic rhinitis in offspring. The aim of this study was to comprehensively evaluate the association between maternal tobacco exposure during pregnancy and allergic rhinitis in offspring by meta-analysis and to provide reference for clinical work.

**Methods:**

Literatures were searched in CNKI, Wanfang Data, VIP, SinoMed, PubMed, Web of science and Embase up to September 30,2020. Screening, inclusion, quality assessment, data extraction and data analysis of the literatures were conducted. Meta-analysis was performed with Revman 5.3 and State15.1 software. Odds ratio (OR) and 95%CI were used as observation indicators.

**Results:**

We had retrieved 16 articles with 22 independent datasets and 11,49,879 sample size. When all the studies were analyzed together, the results showed that maternal smoking exposure during pregnancy would increase the risk of allergic rhinitis in offspring (OR = 1.13, 95%CI:1.02–1.26), especially maternal passive smoking during pregnancy (OR = 1.39, 95%CI:1.05–1.84). But subgroup analysis showed that maternal active smoking during pregnancy was only significantly associated with offspring allergic rhinitis in cross-sectional studies (OR = 1.24, 95%CI:1.07–1.45) and study done in America study (OR = 1.22, 95%CI:1.05–1.42).

**Conclusions:**

Tobacco exposure during pregnancy could increase the risk of allergic rhinitis in offspring. The importance of avoiding prenatal tobacco exposure should be emphasized more for the health of next generation in the public.

## 1 Introduction

Allergic rhinitis is a prevalent and immunoglobulin E mediated non-infectious chronic inflammatory disease, referring to individual nasal mucosa contacting with allergens, and typical symptoms include sneezing, pruritus, rhinorrhea and nasal congestion, can be accompanied by eye symptoms such as itchy eyes, watery eyes, and conjunctival congestion symptoms.^[1]^ About 10% to 25% of general population worldwide suffer from allergic rhinitis^[2–5]^ which is strongly associated with asthma, seriously affecting the quality of our life. In the past several decades, the prevalence has increased in the world. However, the aetiology of allergic rhinitis is not well established.

Tobacco exposure is a common environmental factor. It had been reported as an important factor involving in allergic rhinitis. One meta-analysis has recovered that not active smoking, but passive smoking^[6]^ in general population was associated with the development of allergic rhinitis in the non-pregnant state. Because a large amount of smoke produced in the smoking process causes serious pollution to the working and living environment, which may increase the allergens in the air, including a variety of harmful components such as formaldehyde, acrolein and other irritating compounds can directly stimulate the respiratory mucosa and cause vasospasm and contraction of the nasal mucosa. Long-term effect will lead to damage to the ciliary cells of the nasal mucosa, becoming the inducement of allergic rhinitis and aggravating the condition.^[7]^

Substantial evidence suggests that detrimental environmental factors exposure in early life not only influence prenatal development, but also may produce structural and functional alteration, leading to increased risks of metabolic, cardiovascular, and neuroendocrine disorders in offspring. “Fetal origin theory of adult disease” or “Developmental Origins of Health and Diseases (DOHaD)” theory, has become the foundation for this increasingly popular scientific field.^[8]^ Many studies have demonstrated that prenatal and neonatal factors, such as pregnancy diseases, delivery mode and feeding type may influence the risks of allergic rhinitis in offspring.^[9,10]^ Tobacco exposure is also the most important toxic exposures in utero and in early life, which has been implicated in the aetiology of asthma and some allergic disease in offspring. However, the aforementioned meta-analysis showed that maternal smoking exposure during pregnancy is not associated with the risk of allergic rhinitis in the offspring. But active smoking during pregnancy vs passive smoking during pregnancy or other subgroup analysis had not been considered in the analysis. Furthermore, more and more studies still further focus on this topic and have assessed the association between smoking exposure during pregnancy and allergic rhinitis in the following years. Some studies showed that not only maternal active smoking^[11–14]^ but also maternal passive smoking^[15–17]^ could increase the risk of AR in offspring. However, others have showed conflicting evidence suggesting that both maternal active smoking^[13–16,18–25]^and maternal passive smoking^[26]^ have no effect on the risk of allergic rhinitis in offspring. Hence, published studies have shown conflicting results. Whether smoke exposure in utero is causally linked to the development of offspring allergic rhinitis or not is an unsettled matter. Therefore, it is necessary to summarize all of available evidence and carry out meta-analysis on tobacco exposure during pregnancy, the critical time window, on the risk of allergic rhinitis in offspring. We aim to provide more reference for clinical work of allergic rhinitis management and ensure public health safety.

## 2 Methods

### 2.1 Search strategy

Comprehensive literatures were searched in CNKI (China National Knowledge Infrastructure), Wanfang Data, VIP (China Science and Technology Journal Database), SinoMed, PubMed, Web of science and Embase. We have applied the following algorithm both in medical subject heading and in free text words:(“rhinitis allergic” OR “allergic” OR “allergic rhinitis” OR “hay fever” OR “allergic diseases” OR “hypersensitivity” OR “hypersensitivities” OR “allergy” OR “allergies” OR “allergic reaction” OR “allergic reactions” OR “reaction allergic” OR “reactions allergic”) AND (“pregnancy” OR “pregnancies” OR “gestation” OR “perinatal period”) AND (“offspring” OR “children” OR “child”) AND (“risk factors” OR “risk factor” OR “influence factors” OR “influence factor” OR “relative” AND “risk” OR “relative risk” OR “risks”). To identify all potentially eligible studies, the references lists of all comprised studies were examined. We searched from the beginning of databases up to September 30, 2020. No language restriction of publication was applied.

### 2.2 Inclusion criteria

Studies were selected when:

1. the study type was cohort, case-control or cross-sectional study.
2. the outcome of interest was offspring's allergic rhinitis defined by physician diagnosis, skin prick test (SPT) or international study of asthma and allergies in childhood (ISAAC) questionnaires or questionnaires.
3. the exposure factor was maternal smoking during pregnancy including maternal active smoking and maternal passive smoking.
4. studies offered available data on the relevant risk estimates including odds ratio (OR) and their confidence intervals (CI), or enough data to compute them.

### 2.3 Data extraction and quality assessment

The information of each eligible study was extracted: study design, the name of the first author, year of publication, population origin, diagnostic mode, sample size, children age when investigated, maternal active or passive smoking.

Two grading criteria were used to evaluate the quality of the included literatures. For cohort and case-control studies, the new castle-ottawa scale (NOS)^[27]^ was used. There were 8 items in the NOS and the total score was 9 points, including 4 items (4 points) in the study population selection, 1 item (2 points) in the inter-group comparability and 3 items (3 points) in the measurement of the results. The literature with score ≥ 6 is considered to be high quality and was included (Table 1). For cross-sectional studies, the Joanna Briggs Institute (JBI) Critical Appraisal tool^[28,29]^ was used. This tool has 9 questions, including sampling methods, study subjects, data collection, analysis methods and so on. Answer to the question is “yes”, “no”, “unclear” or “not applicable”. The proportion of the “yes” answer based on the 9 questions was used to determine the final grade for each paper. Literature with total score ≥50% was regarded as a high-quality document and was included.^[30]^

**Table 1:** General information of the included (cohort study and case-control study).

Author	Population origin	Diagnostic mode	Sample size	Children age (y)	Active or passive smoking
Butland 1997^[22]^	Europe	questionnaire	15,564	16	active
Austin 1997^[18]^	Europe	questionnaire	1537	12, 14	active
Magnusson 2005^[23]^	Europe	questionnaire	7844	14–18	active
Johansson 2007^[21]^^∗^^,^^†^	Europe	questionnaire	8850	3	active
Thacher 2014^[19]^	Europe	questionnaire	3798	<16	active
Patil 2015-IoW^[13]^	Oceania	ISAAC	1373	10	active
Patil 2015-FAIR^[13]^	Oceania	ISAAC	969	10	active
Liao 2015^[17]^^∗^	Asia	SPT	19,866	4∼12	passive
Mitselou 2020^[12]^	Europe	from the Swedish NPR^‡^	10,59,600	0–13	active

∗ Case-control study, the others are cohort studies

† Rhinitis and Rhinitis+ running eyes

‡ ISAAC = international study of asthma and allergies in childhood (ISAAC), NPR = National Patient Register, SPT = skin prick test.

Two researchers read through the articles to selected relevant studies, abstracted data and evaluated methodological quality of included studies independently. If no agreement was reached, then a third researcher would help to decide.

### 2.4 Data analysis

The statistical analysis was performed using Stata version 15.1 (StataCorp) and Review Manager 5.3 (Cochrane). The odds ratio (OR) and its 95%CI were transformed to natural logarithms to normalize their distribution, stabilize variances, and it's convenient to compute standard errors. The pooled odds ratio (OR) and its 95%CI for each outcome of interest were calculated by weighting the inverse of variance. Preference should be given to estimates adjusted for potential confounding factors rather than rough estimates. The study heterogeneity was tested by using Cochrane Q test and I^2^statistics. When *P* > .1 and I^2^ < 50%, it shows that the heterogeneity of the studies is not significant, and the fixed effect model is used, whereas the random effect model is used. In addition, subgroup analyses were conducted to explore reasons for heterogeneity based on exposure type (active or passive smoking), study design, population origin, maternal complications. Publication bias was assessed using funnel plot and Begg's rank correlation. Sensitivity analysis was performed by exclude the study one by one. *P* < .05 was deemed statistically significant.

## 3 Results

**Figure 1:**
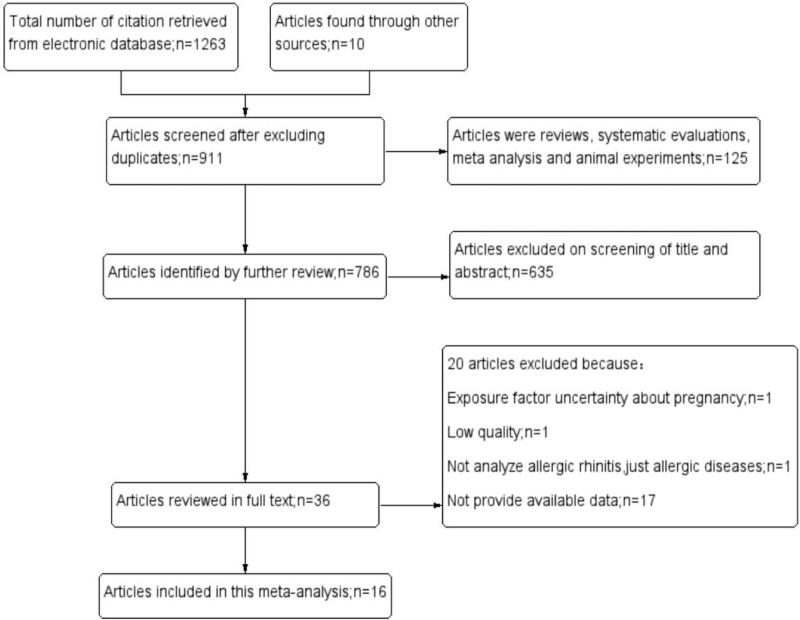
PRISMA flow chart of literature screening.

The flow diagram of the review process is presented in Figure 1. From an initial 1273 citations identified by searching from seven databases and other source, 362 citations were excluded because of duplicates. 125 articles were excluded because they were reviews, systematic evaluation, meta-analysis or animal experiments. 635 articles were excluded by scrutinizing the title and abstract that they did not fulfill the selection criteria.36 articles were reviewed in full text. 20 articles were excluded because 1 article did not specify the smoking exposure time is during pregnancy, 1 article did not specify allergic rhinitis but just mention allergic disease, 17 articles did not provide available data, 1 article was low quality.^[31]^ Finally,16 articles^[11–26]^ that met the inclusion criteria and with high quality were included.

### 3.1 Study characteristics

Study characteristics of the 16 included studies are presented in Tables 1 and 2. Sample sizes varied between 884 to 10,59,600 and total samples size is 1,149,879. The children age varied between 0 to18 years old. Articles are based on 22 independent study populations. With regard to the study type, 6 studies^[12,13,18,19,22,23]^ were cohort studies and 2 studies^[17,21]^ was case-control study. All cohort and case-control studies scored well using the NOS achieving scores between 6 and 9 (Table 3). 8 studies^[11,14–16,20,24–26]^ were cross-sectional study and scored well using the Joanna Briggs Institute (JBI) Critical Appraisal tool (Table 4). With regard to the study region,5 studies^[11,15–17,26]^ were conducted in Asia, 7 studies^[12,18–23]^ in Europe,1 study^[14]^ in North America, 1study^[25]^ in south America,1 study^[13]^ in Oceania and 1^[24]^ in Africa. And among articles, 3 articles^[14,21,25]^ have two definitions of rhinitis. Among the 16 included studies,12 studies^[11–14,18–25]^ with 16 datasets reported maternal active smoking during pregnancy,2 studies^[17,26]^ with 2 datasets reported maternal passive smoking during pregnancy and 2 studies^[15,16]^ with 4 datasets reported maternal both active and passive smoking during pregnancy. Nine^[11,15,18,19,21–24,26]^ of the 16 included studies defined allergic rhinitis through questionnaire, five^[13,14,16,20,25]^ of 16 studies used ISAAC questionnaires. Only one studies^[17]^ used skin prick test for the allergic rhinitis definition, and one article^[12]^ defined allergic rhinitis through the Swedish NPR (National Patient Register).

**Table 2:** General information of the literature was included (cross-sectional study).

Author	Population origin	Diagnostic mode	Sample size	Children age (y)	Active or passive smoking
Villarreal 2003^[14]^^∗^	North America	ISAAC	6749	6–8,11–14	active
Obihara 2005^[24]^	Africa	questionnaire	884	6–14	active
Horak 2007^[20]^	Europe	ISAAC	4109	preschool child	active
Lee 2012–1^[16]^	Asia	ISAAC	7393	≤14	active
Lee 2012–2^[16]^	Asia	ISAAC	7393	≤14	passive
Chen 2012–1^[15]^	Asia	questionnaire	4221	6–9,10–12, 13–15	active
Chen 2012–2^[15]^	Asia	questionnaire	4221	6–9,10–12, 13–15	passive
Azalim 2014^[25]^^†^	South America	ISAAC	1302	child: 6–7, teenager: 13–14	active
Huang 2019^[11]^	Asia	questionnaire	2214	3–6	active
Li 2019^[26]^	Asia	questionnaire	3606	3–6	passive

∗ Rhinitis without flu or cold and rhinitis plus ocular symptoms.

† Rhinitis and rhinitis asthma complications. ISAAC = international study of asthma and allergies in childhood (ISAAC), SPT = skin prick test.

**Table 3:** Quality evaluation of cohort and case-control study.

Author	Study population selection	Intergroup comparability	Outcome measurement	total points
Butland 1997^[22]^	☆☆☆	☆☆	☆☆	7
Austin 1997^[18]^	☆☆☆	☆	☆☆	6
Magnusson 2005^[23]^	☆☆☆☆	☆☆	☆	7
Johansson 2007^[21]^	☆☆☆☆	☆☆	☆☆☆	8
Thacher 2014^[19]^	☆☆☆☆	☆☆	☆☆	8
Liao 2015^[17]^	☆☆☆	☆☆	☆	6
Patil2015^[13]^	☆☆☆☆	☆☆	☆☆☆	9
Mitselou 2020^[12]^	☆☆☆	☆☆	☆☆	7

The New Castle–Ottawa scale (NOS): a scale for assessing the quality of published non-randomized studies in meta-analyses.

**Table 4:** Quality evaluation of cross-sectional study.

Author	1	2	3	4	5	6	7	8	9	Total score
Villarreal 2003^[14]^	√	√	√	√	√	√	?	√	√	89%
Obihara 2005^[24]^	√	√	√	√	√	√	?	√	√	89%
Horak 2007^[20]^	√	√	√	√	√	√	?	√	×	78%
Hugg 2007^[31]^	?	?	?	√	√	√	?	√	×	44%
Lee 2012^[16]^	√	√	√	√	?	√	?	√	√	78%
Chen 2012^[15]^	√	√	√	√	√	×	√	√	×	78%
Azalim 2014^[25]^	√	√	√	√	√	√	?	√	×	78%
Li 2019^[26]^	√	√	√	√	?	×	√	√	?	67%
Huang 2019^[11]^	√	√	√	√	?	×	?	√	×	56%

√: yes; ×: no; ?:unclear; ^∗^: not applicable. 1 Was the sample frame appropriate to address the target population? 2 Were study participants sampled in an appropriate way? 3 Was the sample size adequate? 4 Were the study subjects and the setting described in detail? 5 Was the data analysis conducted with sufficient coverage of the identified sample? 6 Were valid methods used for the identification of the condition? 7 Was the condition measured in a standard, reliable way for all participants? 8 Was there appropriate statistical analysis? 9 Was the response rate adequate, and if not, was the low response rate managed appropriately?

### 3.2 Meta-analysis

**Figure 2:**
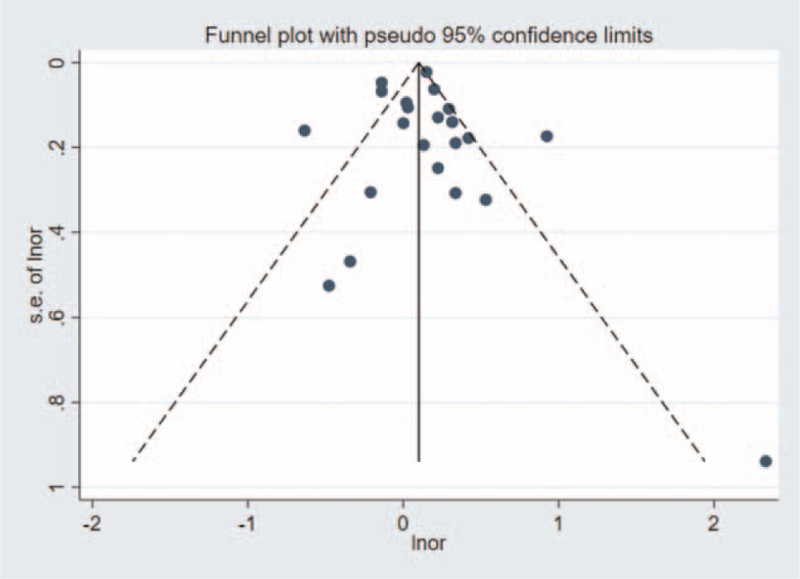
Funnel plot: the influence of maternal exposure to smoking during pregnancy on allergic rhinitis in offspring.

The funnel diagram showed that the left and right distribution of each study is basically symmetrical (Fig. 2) and the Begg's rank correlation test results showed *P* = .978 (>.05), indicating no publication bias in this meta-analysis. Heterogeneity test showed significant heterogeneity among 16 studies (I^2^ = 81%, *P* < .00001), so the random effect model was used for all studies including subgroup analysis.

Results of the meta-analysis including all of the 16 eligible studies with 22 independent datasets showed a significant correlation between maternal exposure to smoking during pregnancy and the risk of allergic rhinitis in offspring (OR = 1.13, 95%CI:1.02–1.26, *P* = .02) (Fig. 3   A). Subgroup analysis showed that maternal smoking during pregnancy was not correlated with offspring allergic rhinitis (OR = 1.04, 95%*CI*: 0.87–1.25, *P* = .65) in case control studies and cohort studies, while maternal smoking during pregnancy was significantly correlated with offspring allergic rhinitis (OR = 1.21, 95%CI:1.09–1.34, *P* < .0002) in the cross section study (Fig. 3   C). And when subgroup analyses based on population origin, there was no significant correlation between maternal smoking during pregnancy and offspring allergic rhinitis in Europe study (OR = 0.94, 95%CI:0.78–1.13, *P* = .52)and in Oceania study (OR = 1.16,95%CI:0.63–2.14, *P* = .63), while there was a significant correlation between maternal smoking during pregnancy and offspring allergic rhinitis in Asia (OR = 1.35, 95%CI:1.05–1.73, *P* = .02) and in America study (OR = 1.22, 95%CI:1.05–1.42, *P* = .01) (Fig. 3   D). Moreover, maternal active smoking was not significantly associated with offspring allergic rhinitis (OR = 1.07, 95%CI:0.95–1.21, *P* = .29), while maternal passive smoking was significantly associated with offspring allergic rhinitis (*OR* = 1.39,*95%CI*:1.05–1.84, *P* = .02) (Fig. 3   B).

**Figure 3:**
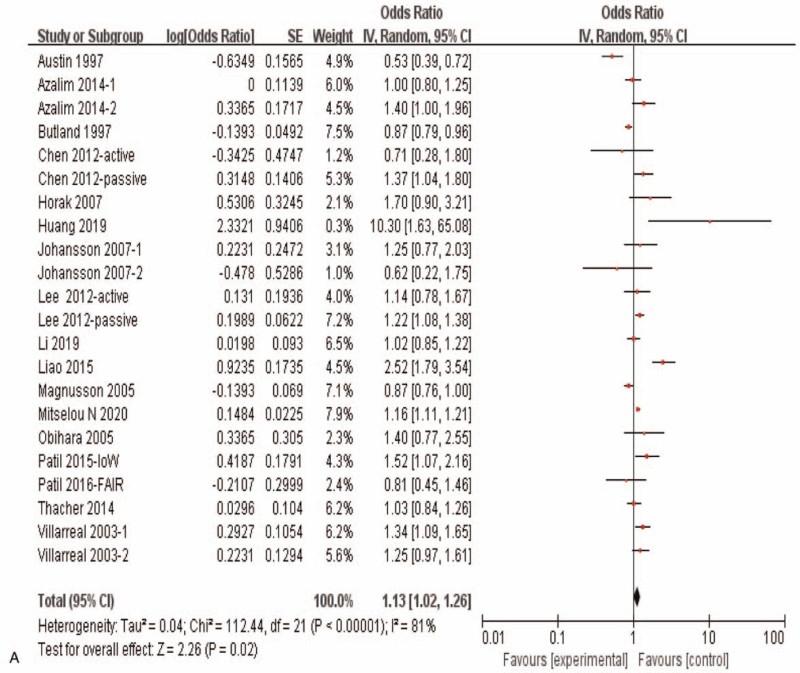
Meta-analysis of the effects of maternal exposure to smoking during pregnancy on offspring allergic rhinitis. All included studies were analyzed. **(A).** All included studies were analyzed. **(B).** Subgroup analysis of maternal active smoking vs maternal passive smoking. **(C).** Subgroup analysis of different research type. **(D).** Subgroup analysis of different study region.

**Figure 3 (Continued):**
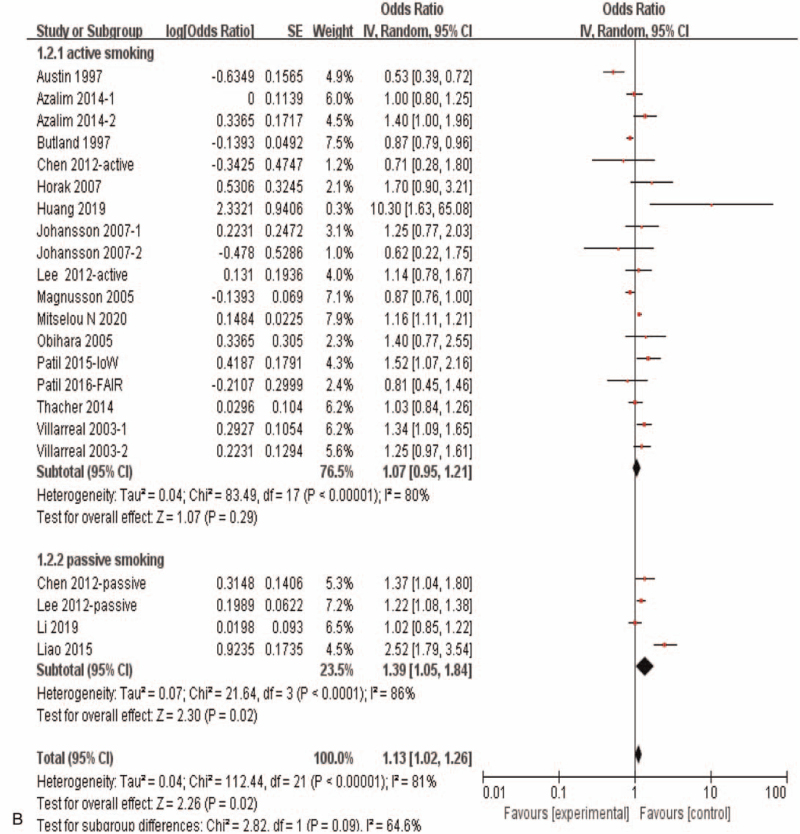
Meta-analysis of the effects of maternal exposure to smoking during pregnancy on offspring allergic rhinitis. All included studies were analyzed. **(A).** All included studies were analyzed. **(B).** Subgroup analysis of maternal active smoking vs maternal passive smoking. **(C).** Subgroup analysis of different research type. **(D).** Subgroup analysis of different study region.

**Figure 3 (Continued):**
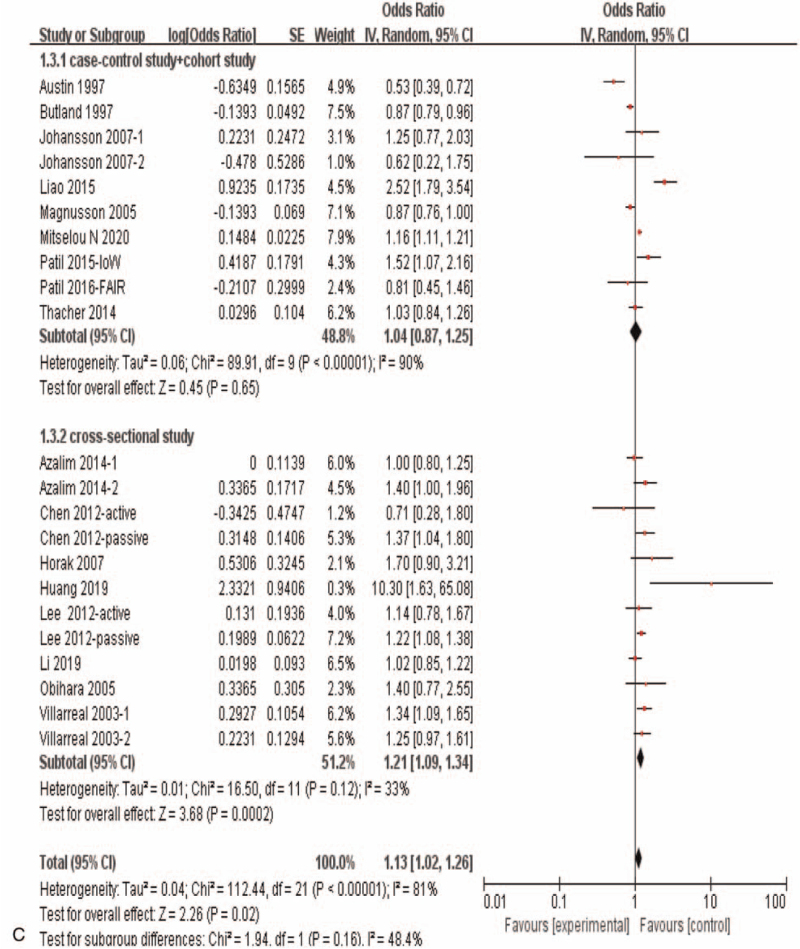
Meta-analysis of the effects of maternal exposure to smoking during pregnancy on offspring allergic rhinitis. All included studies were analyzed. **(A).** All included studies were analyzed. **(B).** Subgroup analysis of maternal active smoking vs maternal passive smoking. **(C).** Subgroup analysis of different research type. **(D).** Subgroup analysis of different study region.

**Figure 3 (Continued):**
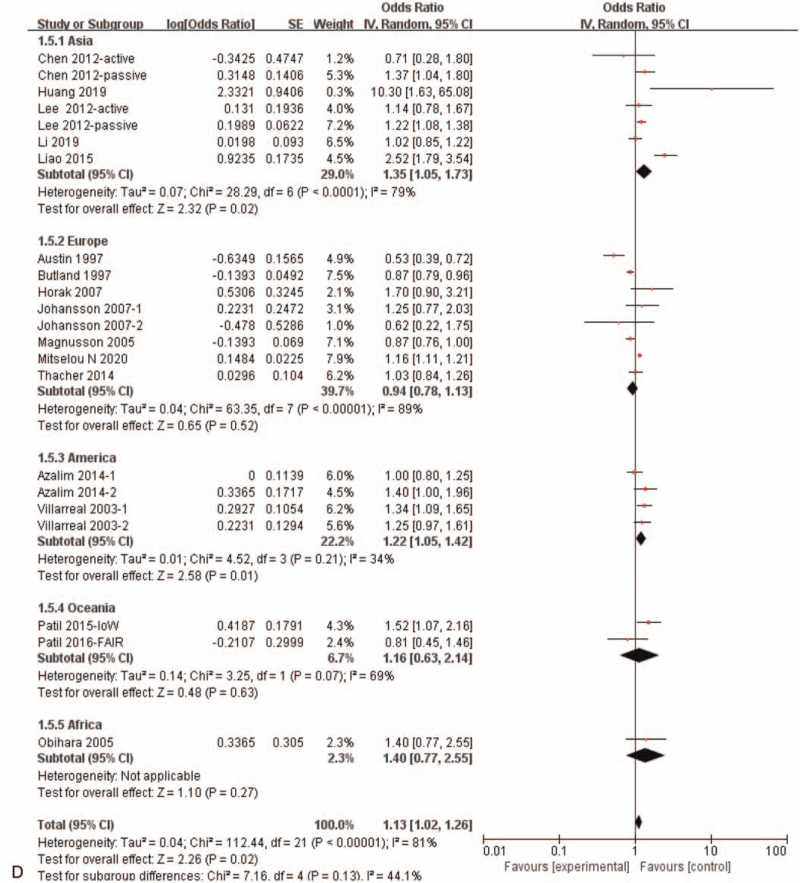
Meta-analysis of the effects of maternal exposure to smoking during pregnancy on offspring allergic rhinitis. All included studies were analyzed. **(A).** All included studies were analyzed. **(B).** Subgroup analysis of maternal active smoking vs maternal passive smoking. **(C).** Subgroup analysis of different research type. **(D).** Subgroup analysis of different study region.

In view of the strong tendency of correlation between maternal active smoking and allergic rhinitis risk in offspring (OR = 1.07, 95%CI:0.95–1.21, *P* = .29), sub-group analyses in this condition was carried out in further, and the results showed that in the cohort study there was no significant correlation between maternal active smoking during pregnancy and offspring allergic rhinitis (OR = 0.94, 95%CI:0.78–1.13, *P* = .50), while in cross-sectional study there was a significant correlation (OR = 1.24, 95%CI:1.07–1.45, *P* = .006) (Fig. 4 A). It showed significant correlation in America study (OR = 1.22, 95%CI: 1.05–1.42, *P* = .01), while no significant correlation in Asia study (OR = 1.43, 95%CI:0.55–3.74, *P* = .47), Europe study (OR = 0.94, 95%*CI*:0.78–1.13, *P* = .52)and Oceania study (OR = 1.16, 95%*CI:*0.63–2.14, *P* = .63) (Fig. 4 B).

**Figure 4:**
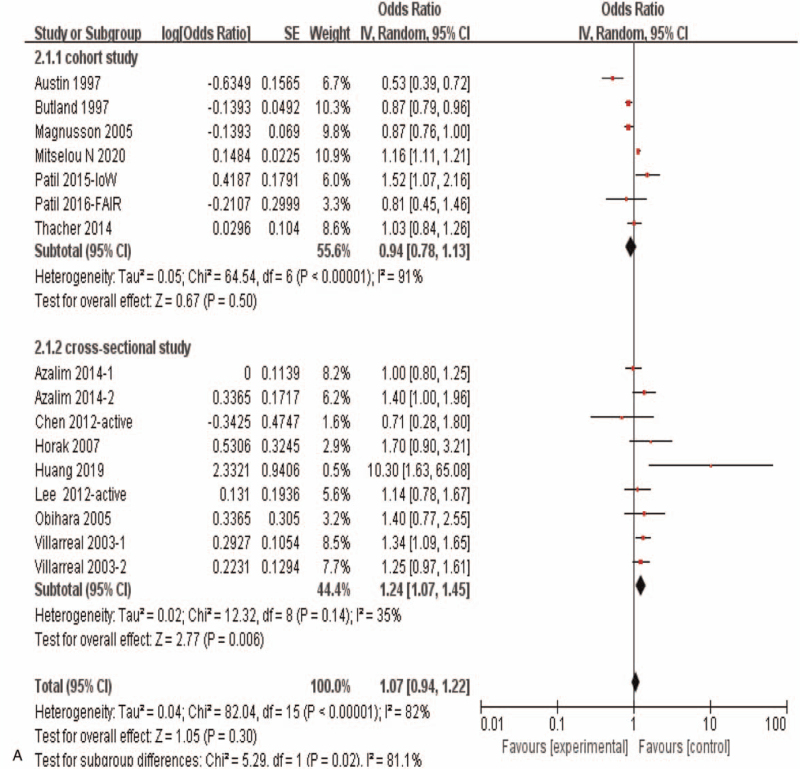
Meta-analysis of the effects of maternal exposure to smoking during pregnancy on offspring allergic rhinitis- under condition with maternal active smoking during pregnancy. **(A).** Subgroup analysis of different research type. **(B).** Subgroup analysis of different study region.

**Figure 4 (Continued):**
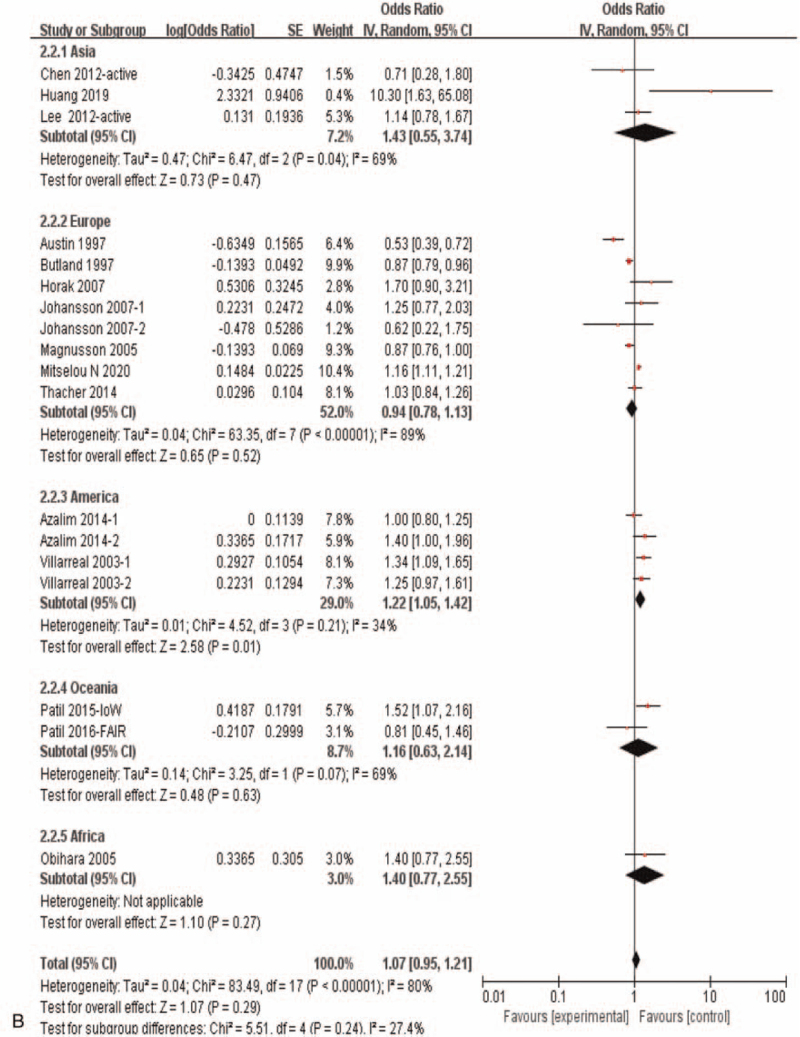
Meta-analysis of the effects of maternal exposure to smoking during pregnancy on offspring allergic rhinitis- under condition with maternal active smoking during pregnancy. **(A).** Subgroup analysis of different research type. **(B).** Subgroup analysis of different study region.

### 3.3 Sensitivity analysis

Removing the included literatures one by one did not affect the results of this study, which means that the above results are stable and reliable.

## 4 Discussion

This meta-analysis summarized all the retrievable articles to date and evaluated smoking exposure during pregnancy and the risk of allergic rhinitis in offspring. The results showed that maternal smoking exposure during pregnancy would increase the risk of allergic rhinitis in offspring, especially maternal passive smoking during pregnancy.

Exposure to environmental tobacco smoke at critical times (i.e. during pregnancy and early life) has been identified as a potential cause of allergic disease in children.^[32]^ A systematic review of 43 studies based on 29 different birth cohorts reported that smoking during pregnancy increased the risk of wheezing in children <6 years of age by 36% (OR, 1.36; 95%CI:1.19–1.55), and increased asthma risk in children ≥6years old by 22% (OR,1.22; 95%CI:1.03–1.44).^[33]^ Chunhong Duan's meta-analysis indicated that maternal smoking during pregnancy could increase the risk of recurrent wheezing in infancy.^[34]^ It is concluded that environmental tobacco smoke exposure increases the risk of wheeze or asthma in children by at least 20%.^[35]^ Smoking during pregnancy and exposure to environmental tobacco smoke in childhood may be non-allergic factors associated with increased risk and incidence of persistent asthma or rhinitis.^[36]^ The result of our meta-analysis and systematic review displayed that active and passive smoking exposure during pregnancy could increase the offspring allergic rhinitis risk by at least 13%. With the previous meta-analysis,^[6]^ which showed that allergic rhinitis was not significantly associated with active smoking (pooled RR = 1.02,95%CI: 0.92–1.15), but was significantly associated with passive smoking (pooled RR = 1.10, 95%CI:1.06–1.15) in the non-pregnant state. Taken together, it is clear that environmental tobacco smoke at anytime would play an important role in the development of allergic rhinitis.

Epidemiological studies have demonstrated that perinatal environmental smoking exposures have adverse effects and are important contributors in the development of offspring asthma or allergic disease. Clinic studies disclosed that fetal exposure to maternal active or passive smoking was related to lower birth weight, which increased the risk for reduced lung function, respiratory tract infections and asthma or wheeze.^[37,38]^ A number of animal studies have been implemented to uncover the mechanism. Rhesus monkeys studies have suggested nicotine exposure during pregnancy as the key factor causing alterations in pulmonary function, possibly because nicotine is transported across the placenta and directly interacts with nicotinic acetylcholine receptors in pulmonary vessels to alter connective tissue expression and cause vascular structural alterations.^[39,40]^ Ferrini's study has revealed that prenatal exposure to environmental tobacco smoke predisposes offspring to an exacerbated allergic airway inflammation, which is associated with a reduction in pulmonary NK cell function.^[41]^ Environmental tobacco smoke exposure during pregnancy is considered as possible factor of the development of childhood allergic diseases through epigenetic mechanisms,^[42–45]^ mainly including histone acetylation, microRNA (miRNA) expression, and DNA methylation. However, the causal relationship between smoking during pregnancy and offspring allergic rhinitis is not well defined.

The strength of our study is that we comprehensively analyzed the relationship between maternal exposure to smoking during pregnancy and the risk of allergic rhinitis in offspring so far. We estimated aggregate data from 22 independent datasets, including 1,149,879 participants. Moreover, the quality of the included literature was generally good. However, the results of this study are subject to several limitations. Firstly, we can’t exclude literatures that may not contain potential confounding factors, such as maternal factors, fetal factors, intrauterine environmental exposure and postnatal environmental exposure that are all directly or indirectly related to the results. Secondly, we found a significant association between smoking during pregnancy and allergic rhinitis in offspring in cross-sectional studies, but not in cohort studies, possibly due to the few cohort studies included. Thirdly, maternal active smoking during pregnancy was not significantly associated with offspring allergic rhinitis in all of included studies (OR = 1.07, 95%CI: 0.95–1.21, *P* = .29), but only significantly associated with allergic rhinitis in their offspring in cross-sectional studies and studies in Asia and America, possibly due to the limitations of the types of included literature and the diversity of the distribution area of the study population. And that many studies have defined allergic rhinitis by questionnaire, which has inherent limitations based on the design, such as the respondents may have memory bias. Therefore, future studies should use more objective methods to diagnose allergic rhinitis, make objective measurements of environmental tobacco smoke, such as measuring nicotine in hair^[46]^ and design more cohort study.

## 5 Conclusions

To sum up, tobacco exposure during pregnancy could increase the risk of allergic rhinitis in offspring involving multi-mechanisms including epigenetic ways. Therefore, mothers should minimize exposure to smoking environment in perinatal time to reduce the influence transferring to next generation. A study^[11]^ showed that maternal and paternal allergy themselves increased the risk of the offspring allergic rhinitis through parental genetic ways. However, researches studying maternal smoking exposure during pregnancy together with maternal and/or paternal with allergic disease meanwhile on next generation are missing up to now. Hence, more works are needed to further investigate the interactions of the parental allergic genetic effect and epigenetic effect of smoking and other hazardous factors on disease risk in offspring.

## Acknowledgments

We would like to acknowledge and thank the team members (Key Laboratory of Model Animals and Stem Cell Biology in Hunan Province, School of Medicine, Hunan Normal University, Hunan 410013, China; Key Laboratory of Molecular Epidemiology of Hunan Province, School of Medicine, Hunan Normal University, Hunan 410081, China) for their help in the codification process of this study

## Author contributions

**Conceptualization:** Yaqian Zhou, Yunpeng Dong.

**Data curation:** Yaqian Zhou, Junrong Chen, Jinhua Shen, Yide Yang.

**Formal analysis:** Yaqian Zhou.

**Funding acquisition:** Jian Li.

**Investigation:** Yaqian Zhou.

**Methodology:** Yaqian Zhou, Yide Yang.

**Supervision:** Jian Li, Yunpeng Dong.

**Validation:** Yunpeng Dong.

**Visualization:** Yunpeng Dong.

**Writing – original draft:** Yaqian Zhou.

**Writing – review & editing:** Mei Tian, Liujiang Song, Jian Li.
